# Are mini multiple gatherings akin to mass gathering events and do they constitute a blind spot in influenza preparedness in post-COVID Europe?

**DOI:** 10.1016/j.nmni.2026.101710

**Published:** 2026-01-22

**Authors:** Giancarlo Ceccarelli, Francesco Branda, Fabio Scarpa, Marta Giovanetti, Gabriella d’Ettorre, Massimo Ciccozzi

**Affiliations:** a Department of Public Health and Infectious Diseases, University of Rome Sapienza, Rome, Italy; b Azienda Ospedaliero Universitaria Umberto I, Rome, Italy; c Genomics, AI, Bioinformatics, Infectious Diseases, Epidemiology Group (GABIE), Rome, Italy; d Migrant and Global Health Research Organization (Mi-HeRO), Rome, Italy; e Unit of Medical Statistics and Molecular Epidemiology, Università Campus Bio-Medico di Roma, Rome, Italy; f Department of Biomedical Sciences, University of Sassari, Sassari, Italy; g Climate Amplified Diseases and Epidemics (CLIMADE), Brazil, Brazil; h Instituto Rene Rachou, Fundação Oswaldo Cruz, Minas Gerais, Brazil; i Sciences and Technologies for Sustainable Development and One Health, Università Campus Bio-Medico di Roma, Italy

**Keywords:** Influenza, COVID-19, Mass gatherings, Distributed mass gatherings, Surveillance, Preparedness

Post COVID-19 preparedness for influenza and other respiratory pathogens in Europe has largely focused on strengthening surveillance systems vaccination strategies and risk assessment frameworks for healthcare settings schools workplaces and formally recognised mass gathering events [1,2]. Classical mass gathering theory is primarily grounded in the co-presence of large numbers of people in a single location for a defined period generating exceptional demands on public health preparedness and response capacities [3]. However the continued emergence of novel and drifted influenza strains exposes a persistent blind spot in these frameworks namely the epidemiological relevance of synchronised private social events that fall outside conventional mass gathering definitions.

We propose the concept of the Distributed Mass Gathering Event (DMGE) to describe the simultaneous occurrence of millions of small predominantly indoor social gatherings that are geographically dispersed yet tightly synchronised in time (Fig. 1). Typical examples include Christmas and New Year family dinners religious feasts or culturally defined festive meals. While none of these events individually meets the numerical or spatial thresholds of a classical mass gathering their temporal synchronisation repetition and cumulative population exposure render them epidemiologically comparable to and in some circumstances more consequential than single large scale events [4,5].

**Fig. 1:**
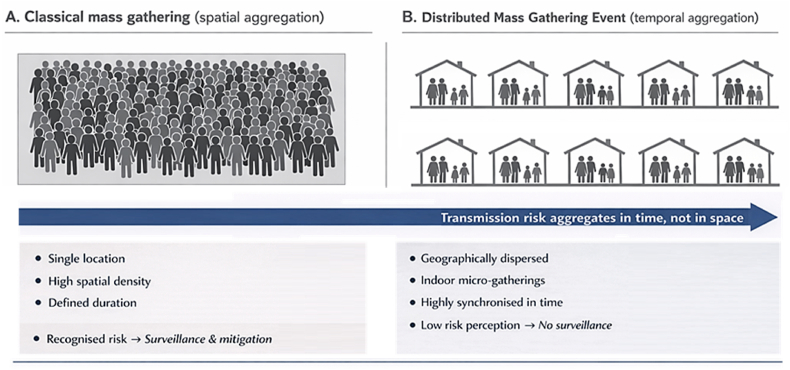
**DMGEsand temporal aggregation of transmission risk.** Panel illustrates a classical mass gathering characterised by spatial concentration of individuals within a single venue, high crowd density, and a clearly defined duration, conditions under which infectious disease risk is readily recognised and typically addressed through dedicated surveillance and mitigation strategies. Panel B depicts a distributed mass gathering event, in which transmission risk emerges from multiple geographically dispersed, predominantly indoor micro-gatherings that are synchronised in time rather than space. In such settings, individual events are often perceived as low risk, resulting in limited or absent public health surveillance. Overall, the figure highlights how, in DMGEs, infectious disease transmission risk aggregates temporally rather than spatially, posing distinct challenges for preparedness, detection, and response.

From a transmission dynamics perspective the defining features of mass gatherings high contact intensity prolonged exposure heterogeneous mixing patterns and reduced sensitivity of routine surveillance are all present in DMGEs albeit in a decentralised form [3,6]. In this context aggregation does not occur in physical space but in time creating a transient yet population wide amplification window for respiratory pathogens. DMGEs can therefore be conceptualised as functionally equivalent mass gatherings whose epidemiological relevance emerges only when assessed at population scale rather than at the level of individual events (Table 1).

**Table 1:** ***Key epidemiological and preparedness differences between classical mass gatherings andDMGEs.****While classical mass gatherings concentrate transmission risk through spatial co-presence and are therefore routinely addressed by public health preparedness and surveillance systems, DMGEs generate epidemiological relevance through temporal synchronisation of millions of small, private, predominantly indoor gatherings. This decentralised configuration renders DMGEs largely invisible to conventional mass gathering frameworks despite their potential to act as population-wide amplification settings for respiratory pathogens.*

DIMENSION	CLASSICAL MASS GATHERING	DMGE
Mode of aggregation	Spatial aggregation of large numbers of individuals within a single, clearly delimited location	Temporal aggregation of a very large number of small-scale gatherings synchronised over a short and predictable time window
Geographical localisation	Single site or limited geographical area (e.g. stadiums, pilgrimage sites, festival venues)	Geographically dispersed settings, predominantly private domestic environments
Typical duration	Hours to a few days, usually corresponding to a single organised event	Several days to weeks, often linked to culturally or religiously defined festive periods
Predominant environment	Mixed indoor–outdoor settings or large ventilated venues	Predominantly indoor, enclosed spaces with limited ventilation
Social mixing patterns	Broad and often heterogeneous mixing, including large numbers of strangers	Repeated, close, and prolonged contact within stable social units, characterised by intense intergenerational mixing
Involvement of vulnerable populations	Variable and often limited due to self-selection or risk avoidance behaviours	Systematic inclusion of older adults, individuals with chronic conditions, and immunocompromised persons within family or household settings
Public health surveillance and preparedness	Actively recognised, assessed, and managed through structured risk assessment, surveillance, and mitigation measures	Largely unrecognised by preparedness frameworks and therefore excluded from dedicated surveillance or mitigation strategies
Risk perception and behavioural response	Generally high, with increased awareness and adoption of preventive behaviours	Generally low, with domestic settings perceived as intrinsically safe and protective
Impact on healthcare systems	Immediate or near-immediate increase in healthcare utilisation temporally linked to the event	Delayed but concentrated increase in hospital admissions, intensive care utilisation, and mortality following the festive period

Recent analyses of respiratory pathogen dynamics support this temporal aggregation model. Silva et al. demonstrated synchronised COVID-19 incidence peaks across Portuguese municipalities driven by concurrent mobility patterns and social activity cycles (including commuting flows, workplace and school attendance, and periodic restriction and reopening transitions), even in the absence of large, centralized gatherings [7]. Li et al. reported that the implementation and subsequent relaxation of public health and social measures in China produced marked temporal shifts in the incidence of 24 notifiable infectious diseases, particularly respiratory and enteric infections. These shifts reflected synchronised behavioral cycles including lockdown reopening transitions, altered mobility, and school and workplace attendance patterns that reshaped disease seasonality without requiring spatial mass gatherings [8]. Together, these findings empirically reinforce the concept that population-wide synchronisation of small, dispersed social gatherings and behavioral cycles can generate epidemic dynamics comparable to those produced by conventional mass gatherings, even in the absence of physical co-location.

In Italy the Christmas period represents a paradigmatic example of a DMGE. Traditional prolonged indoor meals often lasting several hours are characterised by close interpersonal proximity limited ventilation intense vocal interaction and repeated shared dining. These gatherings systematically involve intergenerational mixing which is a well-established driver of influenza transmission [9]. Children who are recognised as key amplifiers of influenza spread due to high contact rates prolonged viral shedding and central social positioning frequently act as focal points of these events [[9], [10], [11]] while older adults who bear the highest burden of severe influenza outcomes are simultaneously present.

Importantly domestic festive gatherings frequently include individuals with chronic diseases or immunocompromised conditions precisely because the home environment is culturally perceived as safe and protective. This perception lowers individual risk awareness and leads to the systematic inclusion of vulnerable people who might otherwise avoid public mass gatherings or crowded venues [12]. DMGEs therefore uniquely concentrate efficient transmitters highly susceptible hosts and prolonged exposure within the same microenvironment without triggering any formal risk mitigation mechanisms.

When a novel influenza strain circulates in a largely immunologically naive population this configuration may increase the potential for private festive gatherings to act as decentralised amplification settings. Alongside well-established explanations including seasonal transmission dynamics, reporting artefacts during holiday periods, and changes in health-care seeking behaviour, the DMGE framework may offer an additional interpretative lens for recurrent post-holiday epidemiological patterns observed across several European countries. These include an apparent reduction in reported case detection during festive weeks followed by increases in hospital admissions, intensive care utilisation, and influenza-associated mortality in January [13,14].

From a preparedness perspective continued reliance on classical mass gathering definitions risks systematically underestimating the earliest drivers of epidemic expansion. While public events are increasingly subject to structured risk assessment and mitigation in the post COVID era DMGEs remain institutionally invisible and therefore excluded from preparedness planning despite their recurrence predictability and scale [9].

The relevance of DMGEs extends beyond the Christmas period. Similar dynamics may occur during other culturally synchronised periods across Europe including Easter family gatherings, Ramadan evening meals, and seasonal wedding celebrations. Although these events are not conventionally classified as mass gatherings they share core epidemiological characteristics when considered collectively namely repeated indoor exposure intense interpersonal interaction and intergenerational mixing occurring within a narrow temporal window [5].

In light of the evidence presented, the recognition of DMGEs as epidemiologically relevant phenomena calls for a recalibration of preparedness frameworks in Europe. While classical mass gatherings are explicitly managed through pre-event risk assessments, health communication strategies, and structured surveillance, DMGEs demand an approach that extends such vigilance into the domestic and community sphere. This requires not only enhanced surveillance capacity but also an anticipatory governance model that aligns public health messaging with predictable behavioral cycles.

Operationally, integrating DMGE awareness into influenza and respiratory pathogen preparedness implies several strategic shifts. First, national and regional health authorities should establish seasonal preparedness calendars that explicitly recognize periods of synchronised private gatherings, such as Christmas, Easter, or Ramadan. In these intervals, vaccination campaigns should be timed to precede the aggregation window, optimizing immunity levels before widespread social interaction. Moreover, surveillance systems should incorporate high-resolution temporal modeling capable of detecting subtle post-festive incidence increases, drawing on spatiotemporal analytics already validated in COVID-19 research [7,8,17].

Risk communication represents a second pillar of this approach. Culturally sensitive campaigns should emphasize the epidemiological relevance of family gatherings without stigmatizing traditional celebrations. Messaging can focus on ventilation, pre-event testing, and protection of vulnerable individuals, measures that are nonintrusive yet effective in reducing within-household transmission. Importantly, hospital preparedness should account for predictable surges in admissions following these periods, adopting dynamic capacity management rather than reactive crisis responses.

Ultimately, the inclusion of DMGEs in public health planning does not require redefining social behaviors but rather recognizing their collective impact on epidemic dynamics. By acknowledging these distributed events as functional equivalents of mass gatherings, policymakers can close a critical preparedness gap and better anticipate the early amplification phases of influenza and other respiratory pathogens. This approach aligns with recent WHO and ECDC calls for community-level risk integration and represents a cost-effective, socially acceptable extension of post-COVID public health vigilance [15,16].

## CRediT authorship contribution statement

**Giancarlo Ceccarelli:** Conceptualization, Formal analysis, Investigation, Writing – original draft, Writing – review & editing, Methodology, Visualization. **Francesco Branda:** Formal analysis, Data curation, Investigation, Writing – original draft, Writing – review & editing. **Fabio Scarpa:** Investigation, Writing – original draft, Writing – review & editing. **Marta Giovanetti:** Investigation, Writing – original draft, Writing – review & editing. **Gabriella d’Ettorre:** Investigation, Writing – original draft, Writing – review & editing. **Massimo Ciccozzi:** Supervision, Validation, Writing – original draft, Writing – review & editing.
